# Thanks to Reviewers

**DOI:** 10.1177/23247096231155228

**Published:** 2023-02-14

The journal sincerely thanks the following individuals who reviewed one or more manuscripts during 2022:

Agarwal, Gaurav

Alagia, Pat

Ali, Sadia

Baer, Stephanie

Beauchamp, Giovanna

Cobos, Everardo

Colburn, Keith

Crosby, James

Crosby, Steven

Das, Samrat

Deane, Kevin

Dimaio, J.

Dutcher, Janice

Eidt, John

Ganti, Shyam

Ghayee, Hans

Gregg, Lucile

Guerrero, Dubert

Hardin, Elizabeth

Harrison, Taylor

Heidari, Arash

Isoardi, Katherine

Jain, Mamta

Jenkins, Leighann

Kulkarni, Anandita

Lange, Richard

Lee, Mark

Liangpunsakul, Suthat

Lingvay, Ildiko

Lo, Tze Shien

Luby, James

Lyon, Priscilla

Maalouf, Naim

Mcnutt, Markey

Mcphaul, Christopher

Mcphaul, Michael

Means, Robert

Morgan, Bill

Oates, Jim

Pant, Chaitanya

Racke, Michael K.

Rockey, Don

Sakhaee, Khashayar

Schivo, Michael

Simha, Vinaya

Southern, Paul

Stecker, Mark

Suvannasankha, Attaya

Tanpaiboon, Pranoot

Tessnow, Alex

Trandafirescu, Theo

Van Buren, Peter

Vasquez, Miguel

Vernino, Steven

Wiernik, Peter

Wiley, Zanthia

Win, Theingi Tiffany

Zuckerman, Marc

